# Effect of the Rearing Substrate on Total Protein and Amino Acid Composition in Black Soldier Fly

**DOI:** 10.3390/foods10081773

**Published:** 2021-07-30

**Authors:** Andrea Fuso, Silvia Barbi, Laura Ioana Macavei, Anna Valentina Luparelli, Lara Maistrello, Monia Montorsi, Stefano Sforza, Augusta Caligiani

**Affiliations:** 1 Food and Drug Department, University of Parma, Via Parco Area delle Scienze 17/A, 43124 Parma, Italy; andrea.fuso@unipr.it (A.F.); annavalentina.luparelli@unipr.it (A.V.L.); stefano.sforza@unipr.it (S.S.); 2 Interdepartmental Research Center for Industrial Research and Technology Transfer in the Field of Integrated Technologies for Sustainable Research, Efficient Energy Conversion, Energy Efficiency of Buildings, Lighting and Home Automation—En&Tech, University of Modena and Reggio Emilia, Via Amendola 2, 42122 Reggio Emilia, Italy; s.barbi@unimore.it; 3 Department of Life Sciences, University of Modena and Reggio Emilia, Via Giovanni Amendola 2, 42122 Reggio Emilia, Italy; lauraioana.macavei@unimore.it (L.I.M.); lara.maistrello@unimore.it (L.M.); 4 Department of Sciences and Methods for Engineering, University of Modena and Reggio Emilia, Via Giovanni Amendola 2, 42122 Reggio Emilia, Italy; monia.montorsi@unimore.it

**Keywords:** *Hermetia illucens*, insect rearing, vegetable leftovers, protein fraction, amino acids composition, growth substrate

## Abstract

Insects are becoming increasingly relevant as protein sources in food and feed. The Black Soldier Fly (BSF) is one of the most utilized, thanks to its ability to live on many leftovers. Vegetable processing industries produce huge amounts of by-products, and it is important to efficiently rear BSF on different substrates to assure an economical advantage in bioconversion and to overcome the seasonality of some leftovers. This work evaluated how different substrates affect the protein and amino acid content of BSF. BSF prepupae reared on different substrates showed total protein content varying between 35% and 49% on dry matter. Significant lower protein contents were detected in BSF grown on fruit by-products, while higher contents were observed when autumnal leftovers were employed. BSF protein content was mainly correlated to fibre and protein content in the diet. Among amino acids, lysine, valine and leucine were most affected by the diet. Essential amino acids satisfied the Food and Agricultural Organization (FAO) requirements for human nutrition, except for lysine in few cases. BSF could be a flexible tool to bio-convert a wide range of vegetable by-products of different seasonality in a high-quality protein-rich biomass, even if significant differences in the protein fraction were observed according to the rearing substrate.

## 1. Introduction

By 2050, the world will host more than 9 billion people, and the availability of proteins is the main concern in feeding the increasing population, as already pointed out as long ago as 1975 by Meyer-Rochow [1]. Presently, in Western diets the proteins are predominantly introduced through products of animal origin, although the zootechnical production constitutes an important issue from an ecological point of view due to its impact on the environment [2]. A valuable and more sustainable source of protein is represented by insects. They have a high nutritional value [3,4,5] and, compared with traditionally farmed animals, insects have a much higher conversion efficiency and require much less water [2], and their rearing involves much less greenhouse gas emissions [6].

Nowadays, more than one-third of the edible parts of food produced gets lost or wasted; insects could bear an important role in managing and valorizing food waste, since they can be reared on a large variety of bio-waste substrates [7,8], thus contributing to their mass reduction and preventing unnecessary waste of resources and further emissions of greenhouse gas [9]. Therefore, insects fit perfectly in the perspective to valorise bio-waste to create a sustainable food and feed production system that embraces the concept of circular economy and increased sustainability [10,11]. The ability of insects to convert waste materials into high-quality nutrients has long been known [12], and the Black Soldier Fly (BSF, *Hermetia illucens* L., *Diptera, Stratiomiyidae*) is known as one of the most efficient bioconverters among insects, being able to reduce the weight of organic waste up to 75% and converting it into a biomass rich in proteins and lipids [13]. This makes the BSF suitable to be used as feed for farmed animals [14], for biodiesel production [15] and also for cosmetics or pharmaceuticals industries, thanks to its high chitin content [16]. All these applications could be simultaneously tackled through an appropriate fractionation method [17]. Last but not least, the residual larval frass can be employed as a quality soil improver [18,19].

Many studies have shown that BSF larvae can live on many substrates with different characteristics, ranging from mushrooms [20] and winery by-products [21], restaurant waste [8], municipal waste [22], animal manure [10,23] and human faeces [24]. However, according to European legislation (Regulation (EC) No. 1069/2009), when invertebrates are industrially reared, they are considered as “farmed animals”; therefore, the use of animal manure, catering waste or former foodstuffs containing meat and fish as feeding substrates is totally forbidden [25,26]. As a consequence, despite the lower content of nutrients, vegetable and fruit leftovers are increasingly used as rearing substrates for BSF, also due to their high availability in industrialized regions that are fully compliant with the legislation on feed for farmed animals [9]. European Food Safety Authority (EFSA) highlighted that no additional microbiological risks are expected for insect rearing on authorized substrates with respect to other animal farming [26]. In addition, a few studies demonstrated that the eventual pesticides and mycotoxins do not accumulate in BSF biomass after the breeding process [27,28,29,30].

An important point in the use of vegetal substrates as feed for insects is their seasonal availability. As a matter of fact, most of the vegetal leftovers are not constantly available throughout the year, thus it is important, both from the economic and technical point of view, to understand the feasibility of rearing BSF on a variety of substrates and a combination thereof, in order to have a constant BSF production and composition throughout the year.

Many authors have studied the influence of rearing substrates on BSF protein fractions in terms of total amount and of amino acid profile. The protein amount showed marked differences, varying from 32 to 58% on dry matter [13], while for the amino acid composition, studies in the literature are often inconsistent, and this is true for other insect species as well [31]. According to Newton et al., BSF larvae reared on animal manure were lacking some essential amino acids, such as cysteine, methionine and threonine [12], whereas according to Liland et al., BSF larvae had sufficient threonine amounts when reared on a conventional diet supplemented with increasing quantities of seaweed [32]. When fed on a vegetable mix, BSF showed high amounts of aspartic acid, glutamate and arginine [7], but also of leucine [8].

In this paper, a wide range of data was collected on the content and quality of BSF prepupae protein in relation to the rearing substrates used. A total of 49 different combinations of vegetable rearing substrates were tested in order to obtain the most complete picture of the effect of diet on BSF proteins and on the possibility of using diverse substrates for their rearing. All these vegetable by-products have been chosen focusing on their availability in a specific region (Regione Emilia-Romagna, Italy) and mostly on their seasonal availability. The diets were formulated according to a Design of Experiment approach (Mixture Design) based on the nutrient composition of each substrate and their seasonal availability, as reported in Barbi et. al, in the optic of a possible scale-up application of BSF rearing on vegetable by-products across the whole year [33]. To put this approach into practice and fulfil the requirements of a circular economy, it is of primary importance to verify the composition of BSF reared on the different substrates, and in particular their protein fraction that nowadays is considered as the most valuable component of insects.

## 2. Materials and Methods

### 2.1. Materials

The vegetable by-products were collected from different suppliers in the Emilia Romagna Region (northern Italy): Agribologna (Bologna, Italy), Conserve Italia (Bologna, Italy) and Cooperativa Agricola Brisighellese (Ravenna, Italy). The by-products were stored at −20 °C until use, and further grinded with IKA A10 laboratory grinder.

Chemicals: an AccQ-Fluor reagent kit was obtained from Waters (Milford, MA, USA). DL-norleucine, amino acid standard mixture, L-tryptophan, 5-Methyl-DL-tryptophan, L-Cysteic acid, DL-Dithiothreitol, Ethylenediaminetetraacetic acid (EDTA) and Tris-HCl were purchased from Sigma-Aldrich (St. Louis, MO, USA). Sodium dodecyl sulfate was purchased from Biorad (Hercules, CA, USA). All the other solvents, salts, acids and bases were of analytical grade and were purchased from Sigma-Aldrich or Carlo Erba (Milan, Italy).

### 2.2. Insect Rearing Conditions and Substrate Selection

BSF prepupae for the analyses were obtained from a series of experiments carried out in the Applied Entomology laboratory of the University of Modena and Reggio Emilia (Italy). Leftovers selection, rearing substrates design and larvae rearing experiments have been managed by the University of Modena and Reggio Emilia (Italy), as discussed in another study [33]. Essentially, BSF larvae were reared under controlled conditions (27 ± 0.5 °C, 60–70% Relative Humidity, RH) in various mixtures of fruit and vegetable by-products with a different seasonal availability (All-year, Summer and Autumn, Table S1), and the mixtures were designed through a Mixture Design approach. Details on the chemical composition of the substrates are reported in Table S1. The “Gainesville House Fly” diet (50% wheat bran, 30% alfalfa meal and 20% corn meal) [34] was used as the control diet (CTR). Forty-nine experiments were conducted, inoculating exactly 300 BSF larvae (of second and third stage) for each substrate. The rearing experiments was conducted for a maximum period of 65 days, checking regularly the prepupae developed. At each control, the prepupae observed were collected, killed by freezing and stored at −20 °C until further analysis.

### 2.3. Proximate Composition of Rearing Substrates and BSF Prepupae

Proximate composition of agri-food leftovers was determined using standard procedures [35]. Moisture was determined in an oven at 105 °C for 24 h. Crude fat content was determined using an automatized Soxhlet extractor (SER 148/3 VELP SCIENTIFICA, Usmate Velate, Italy) using diethyl ether. Total ash was determined after mineralization at 550 °C for 5 h. Total nitrogen was determined with a Kjeldahl system (DKL heating digestor and UDK 139 semiautomatic distillation unit, VELP SCIENTIFICA) using 6.25 as the mean nitrogen coefficient conversion for vegetable rearing substrates. Dietary fibres of vegetable by-products were determined through the official method AOAC 991.43, while total polyphenol content was determined by using the Folin–Ciocalteu method. Digestible carbohydrates were determined by difference.

Regarding BSF prepupae, the total protein content was determined by the Kjeldahl method, utilizing 4.67 as the nitrogen to protein conversion factor, in order to exclude chitin contributing to total nitrogen, as previously reported [36].

### 2.4. Amino Acid Profile of BSF Prepupae

#### 2.4.1. Sample Preparation

The total amino acid profile was evaluated according to the protocol proposed by Caligiani et al. with some modifications [17]. An amount of 0.5 g of BSF prepupae was hydrolysed with 6 mL of HCl 6 N at 110 °C for 23 h, then the internal standard (7.5 mL of 5 mM Norleucine in HCl 0.1 M) was added. Cysteine was determined as cysteic acid after performic acid oxidation followed by acid hydrolysis. In this case, an amount of 0.5 g of BSF was added to performic acid freshly prepared (by mixing 9 volumes of formic acid with 1 volume of hydrogen peroxide) and samples were kept in an ice bath for 16 h at 0 °C. Then, 0.3 mL of hydrobromidric acid was added and the bromine formed was removed under nitrogen flow. Then, acid hydrolysis was performed as described above.

#### 2.4.2. UPLC/ESI-MS Analysis

The hydrolysed samples were analysed by ultra-performance liquid chromatography with electrospray ionization and mass spectrometry detector (UPLC/ESI-MS, WATERS ACQUITY) after derivatization with 6-aminoquinolyl-N-hydroxysuccinimidyl carbamate (AQC). In particular, UPLC/ESI-MS analysis was performed by using an ACQUITYUPLC separation system with an Acquity BEH C18 column (1.7 μm, 2.1 × 150 mm). The mobile phase was composed of H_2_O + 0.2% CH_3_CN +0.1% HCOOH (eluent A) and CH_3_CN + 0.1% HCOOH (eluent B). Gradient elution was performed: isocratic 100% A for 7 min, from 100% A to 75.6% A and 24.4% B by linear gradient from 8 to 28 min, isocratic 100% B from 29 to 32 min, isocratic 100% A from 33 to 45 min. The flow rate was set at 0.25 mL/min, injection volume 2 μL, column temperature 35 °C and sample temperature 18 °C. Detection was performed by using Waters SQ mass spectrometer: the ESI source was in positive ionization mode, capillary voltage 3.2 kV, cone voltage 30 V, source temperature 150 °C, desolvation temperature 300 °C, cone gasflow (N2) 100 L/h, desolvation gas flow (N2) 650 L/h, full scan acquisition(270–518 *m*/*z*) and scan duration 1 s. Calibration was performed with standard solutions prepared mixing norleucine, amino acids hydrolysate standard mixture, cysteic acid and deionized water.

#### 2.4.3. Tryptophan Determination

Total tryptophan was determined following the protocol proposed by Delgado-Andrade et al. with some modifications [37]. An amount of 0.2 g of sample was weighed and dissolved in 3 mL of 4N NaOH. An amount of 150 µL of 5-methyl-tryptophan (16 mg/100 mL), used as internal standard, was added and mixed. Hydrolysis was then carried out for 18 h at 110 °C. The hydrolysates were aerated and cooled, then carefully acidified to a pH 6.5 with HCl 37%, diluted to 25 mL with sodium borate buffer (0.1 M, pH 9.0) and allowed to stand for 15 min. Samples were finally centrifuged at 4000 rpm for 5 min and supernatant filtered through a 0.45 μm nylon filter membrane into UPLC vials. UPLC/ESI-MS analysis was performed as for the other amino acids.

### 2.5. Statistical Analysis

All analyses on prepupae were carried out in duplicate. Data are expressed as the mean ± standard deviation. Protein and amino acid data were subjected to one-way analysis of variance (ANOVA) followed by a Tukey post hoc test using IBM SPSS software version 21.0 (SPSS Inc., Chicago, IL, USA) to determine differences between samples. Significant differences were compared at a level of *p* < 0.05.

The Pearson correlation coefficient was calculated to evaluate the linear correlations between the nutrients in rearing substrates and protein amounts in BSF prepupae biomass. Both values were taken in absolute terms, calculated according to the following equations:

$$\mathrm{BSF}\;\mathrm{total}\;\mathrm{protein}\;\mathrm{content}=\frac{\mathrm{Protein}\%*\mathrm{total}\;\mathrm{grams}\;\mathrm{of}\;\mathrm{prepupae}\;\mathrm{at}\;\mathrm{the}\;\mathrm{end}\;\mathrm{of}\;\mathrm{the}\;\mathrm{breeding}}{100}$$

$$Leftovers\;total\;nutrient\;content=\frac{\mathrm{nutrient}\%\;\mathrm{in}\;\mathrm{diet}*\mathrm{total}\;\mathrm{grams}\;\mathrm{of}\;\mathrm{diet}}{100}$$

The second equation was applied separately for each nutrient present in the diet: proteins, lipids, fibres, carbohydrates, ashes and polyphenols.

Principal Component Analysis (PCA) was carried out on amino acid data using IBM SPSS software version 21.0 (SPSS Inc., Chicago, IL, USA). PCA was performed through a 52-point matrix (49 samples plus 3 replicates of the control substrate, one for each seasonal period) and 19 variables (18 amino acids and total protein content), and the principal components were derived with the correlation method. As an unsupervised learning approach, PCA allows us to describe the variation in the dataset. Data were visualised by plotting the score plot and the loading plot, the latter allowing the identification of the amino acids having influences on specific grouping of samples according to the rearing substrate.

The Design Expert 12.0 (Stat-Ease) code was used both to set up the experimental plan and to analyse the results. A mixture design was selected to obtain predictive reliability on the effect of the leftovers’ composition on the amino acid content of BSF’s prepupae. Six factors were considered: proteins, fibres, carbohydrates, polyphenols, lipids, and ashes, from which the experimental plan of Table S1 was derived. ANOVA was employed to estimate the influence of each factor over the responses observing the *p*-values (α-level of 0.05) and F-tests. The quality of the fit in terms of regression analysis and the predictive power of the model were evaluated by using the R2 and Pred-R2, respectively. R2 is the proportion of the dependent variable’s variance predictable from the independent variables. In a similar way, Pred-R2 shows how well the model can predict the responses for new observations. Response contour plots were used as functional tools in explaining graphically the role of the main components on the final considered properties [38].

## 3. Results and Discussion

### 3.1. Rearing Substrate Composition and Related BSF Biomass

The composition of rearing substrates, both in terms of leftover combinations and specific nutrient composition is reported in Table S1, while in Table 1, the mean composition of the diets according to the seasonality (substrates available all year, substrates available in summer and substrates available in autumn) is reported and compared with the BSF standard diet (Gainsville diet, CTR). The three groups of tested diets differed between each other and in respect to the control diet. The differences in diet compositions are related to the vegetable by-products employed: (i) fruit peels and pulp (exotic fruits, apple, kiwi, pineapple, melon) for the All-Year diets, (ii) tomato peels/seed and peach pulp/peels for the Summer group and (iii) legume, corn and olive pomace for the Autumn group, although the exotic fruits and melon, as substrates that are available all year round, were introduced in some diets of the AUTUMN and SUMMER groups. Corn, wheat brans and alfalfa were instead the ingredients of the control diet (CTR diet), used as the BSF rearing substrate in the lab colony.

The different ingredients employed in the three groups are also related to a different proximate composition of the diets, as reported in Table 1. The All-Year diets are rich in available carbohydrates and poor in protein and lipids, while the Summer and Autumn groups contain larger amounts of fibres and protein, the latter especially represented in the Autumn group.

Despite differences in composition, all the diets tested allowed BSF to grow (more than 90% of the initial 300 BSF larvae reached the prepupal stage in all experiments), even if with consistent differences in total weight of final prepupae biomass. In Table 1, the mean amount of prepupae (g) obtained for each different rearing mixture is also reported, clearly outlining the prominent effect of seasonality on the BSF prepupae total biomass amount. The specific amount of prepupal biomasses in each rearing experiment is reported in Table S1 of the Supplementary Materials.

As a consequence, the BSF prepupae obtained can be roughly divided into three groups according to their biomass weight at the end of the growing process, which basically correspond to the three groups of vegetable diets administered, classified according to their seasonality. Indeed, the lower quantity of prepupal biomass were obtained by using the All-Year substrates, the intermediate weight with Summer substrates (both of them lower than when using the control diet), while the higher quantities were obtained by employing the Autumn group substrates, which seems to be the most suitable feeding substrate group to maximize the BSF biomass.

### 3.2. Effect of the Rearing Substrate Composition on BSF Prepupae Total Protein Content

Given that the composition of the diets influences the growth of the prepupae, the target of this work, as previously stated, was to verify how the different substrates, and in turn the different growth performance of prepupae (low, medium and high biomassweights), influence the final BSF protein quality and its total amount. BSF samples were clustered on the basis of the prepupal biomass, corresponding to the three groups of BSF diets (All-Year, Summer and Autumn).

**Figure 1:**
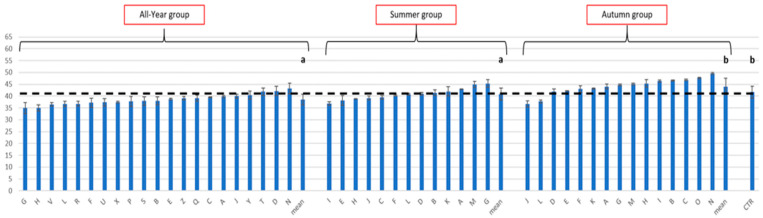
Total protein content (Nx4.67, g/100 g DM) for each BSF sample grown on the 49 diets considered and compared with the control samples (dotted line). Each datum is the mean of two replicates. Global mean of each group was compared with one-way ANOVA (*p*-level 0.05). Different letters (**a**,**b**) on the bars indicate significantly different values.

The total protein content of each BSF prepupa reared on the different substrates is reported in Figure 1 (see Table S1 of the supplementary material for the details about substrate typologies, composition and sample codes). Protein amount, calculated as g/100 g of BSF on dry matter (% DM), varied in the range of 35% to 49.5%. These values agree with the studies in the literature, reporting a range of 32–58% [13].

In general, most of the samples from the All-Year and Summer groups contain lower amounts of protein compared to the control group, although with a few exceptions (Sample N—All-Year, sample A, M, G—Summer). On the contrary, many of the Autumn group samples contain higher amounts of protein with respect to the control, indicating that a better growth performance, as expected, also corresponds to a higher protein content

Taken as a whole (mean), the Autumn group did not show a significant difference with respect to the control, outlining that the agrifood leftovers used in this group could fully replace the control diet, while in the All-Year and the Summer groups, a significantly lower amount of protein was recorded (one-way ANOVA, Tukey post hoc test, *p*-level = 0.05). This indicates that, while growing on these substrates is certainly possible, it would be less efficient and result in a smaller overall amount of protein. Thus, growing on these substrates should be performed by carefully balancing the advantage of re-using leftovers with the disadvantage of having a slightly lower amount of protein.

To better understand which relationship exists between the various diets administered and the production of protein in the prepupae biomass, we correlated the latter and the single nutrients of the diets through linear correlation analysis.

A positive, although moderate, correlation was observed between the lipid content of the diet and the protein content of prepupae (R = 0.54; *p* < 0.001), while non-structural carbohydrates (digestible carbohydrates) showed a moderate negative one (R = −0.56, *p* < 0.001). On the contrary, polyphenols (R = 0.39, *p* < 0.001) and ashes (R = 0.17, *p* = 0.37) in the diet did not affect the protein content of BSF prepupae.

**Figure 2:**
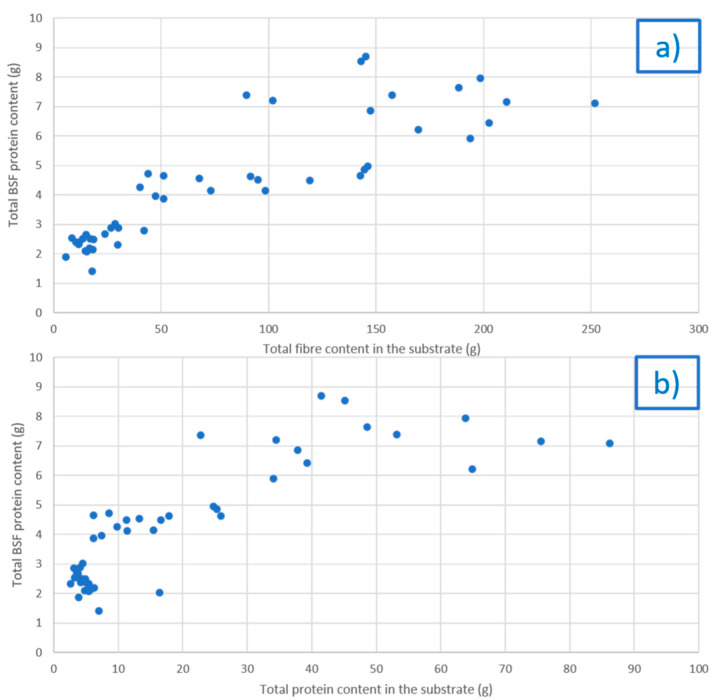
Correlation between total protein content (absolute amount) in the BSF prepupae biomass recorded in each experiment (starting from 300 BSF larvae) and (**a**) total fibre and (**b**) total protein contained in the rearing substrate (absolute amount).

A significant positive correlation was detected between the BSF prepupae protein content and the fibres content of the diet (R = 0.87, *p* < 0.001, Figure 2a). Fibres are typically difficult degrade for most insects, but this positive correlation could be the result of the increased availability of nutrients due to the action of microorganisms able to hydrolyse cellulose, both endogenous and exogenous (i.e., already present in the rearing substrates) [21,39,40,41]. Indeed, previous studies have identified cellulase genes in the gut microflora of BSF larvae [40,42]. Thus, a likely hypothesis for the correlation found might be that the ability of using more and better fibre biomass leads to better larval growth, which in turn also means a higher amount of protein produced.

A positive correlation was also identified between the BSF prepupae protein content and the diet’s protein content in absolute terms (R = 0.84, *p* < 0.001, Figure 2b), confirming previous findings [21,23].

Observing qualitatively the data in Figure 2a,b, a lack of a linear correlation (a plateau is reached) seems evident, especially when a high amount of fibre or protein was present in the diet, suggesting that there is a specific level of these nutrients in the substrates allowing them to reach the maximum amount of protein in insects.

**Figure 3:**
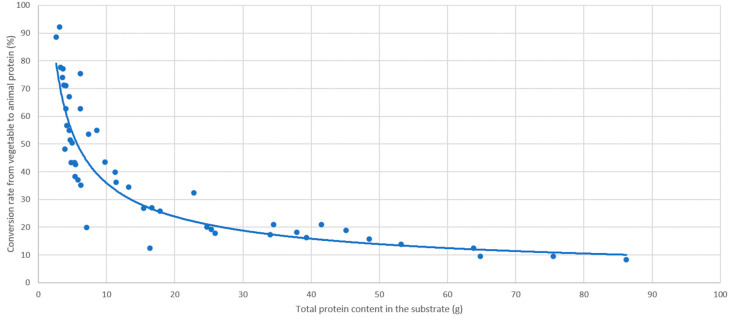
Conversion rate (%) of vegetable protein present in the substrate into BSF protein (absolute amounts, starting from 300 BSF larvae).

More specifically, our data show that by increasing the amount of vegetable protein in the diet, BSF larvae convert progressively a smaller part of it into their own animal protein. In fact, whilst for low-protein diets they were able to convert more than 90%, for the most protein-rich diets, this percentage dropped to 10% (Figure 3). This suggests that for BSF larvae growth and protein content, the amount of protein in the rearing substrate is very important until a minimum threshold is reached, and then it becomes less relevant. These experimental data are in agreement with a recent work about digestive enzyme expression and production in BSF [43]. In this paper, the authors clearly demonstrate that the midgut of *H. illucens* larvae is able to adapt to diets with different nutrient content; an increase in proteolytic activity together with a decrease in α-amylase and lipase activity was observed as a consequence of nutritionally poor diet. Moreover, Barragán Fonseca (2018) demonstrated that larvae feeding on substrates rich in protein have a higher lipid content, and thereby a reduced protein content (in % dry mass) [44]. Consequently, in order to obtain prepupae with a high protein content, according to Figure 3, a good compromise to maintain an advantageous conversion rate would be to rear them on a substrate containing 7% by weight of protein. This value is also in accordance with a previous study [31].

### 3.3. BSF Amino Acid Content

**Figure 4:**
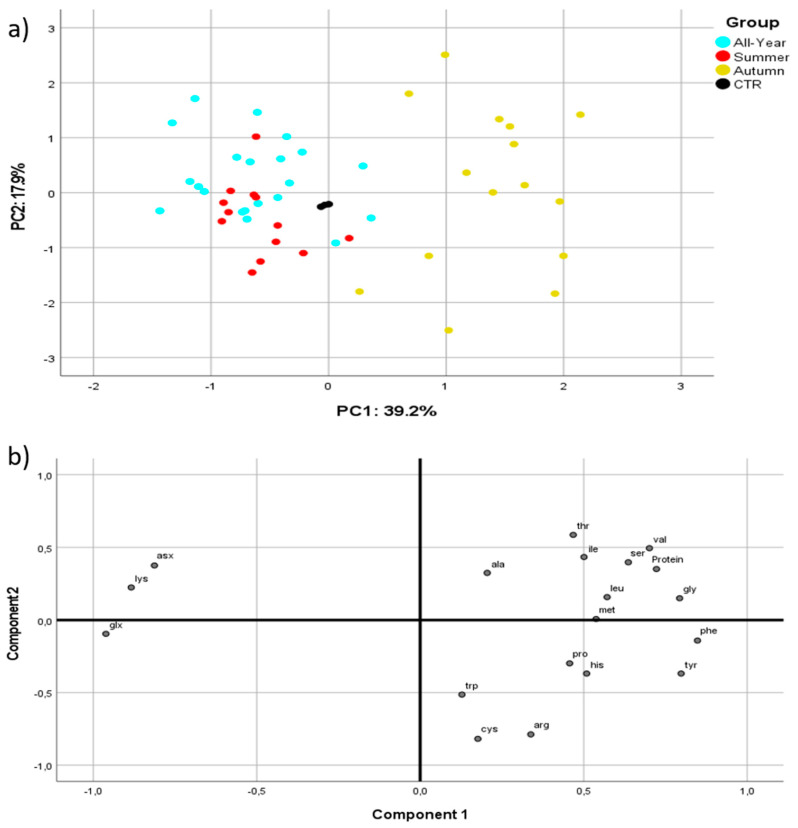
(**a**) Score plot of BSF samples on the first two principal components; (**b**) loadings values of the variables associated with the first two principal components.

To better verify the influence of the rearing substrate composition on the BSF protein nutritional value, further insights into the complete amino acid profile were provided. As a matter of fact, information on the nutritional quality of proteins, and therefore on their amino acid composition, is of utmost importance to understand the possible uses of the BSF protein fraction. Results on the complete total amino acid profile of the BSF prepupae are reported in Table S2. An explorative Principal Component Analysis (PCA) was performed to assess if and how different rearing substrates would affect amino acid composition. Principal Components are new variables obtained by linear combinations of the original variables (amino acids and total protein), allowing us to describe the system variability using only a few components, thus reducing the complexity, enabling the visualization of the samples in a two-dimensional graph. The analysis showed that about 39% of the total variation is explained by the first component (PC1), 57% by the first two components and 69% by the first three components. The most important variables for each principal component are reported in Table S3 of the Supplementary Materials. Figure 4a shows the scatter plot of the scores of PC1 versus PC2. The loadings for the first two components are schematized in the component plot (Figure 4b). Glutamic acid/glutamine, lysine, phenylalanine, aspartic acid/asparagine, tyrosine, glycine, valine, serine and histidine turned out to be the most influencing variables for PC1, PC2 was predominantly characterized by cysteine, tryptophan, arginine and threonine, while leucine, isoleucine and methionine had the greatest effect for PC3. Figure 4a shows a partial separation of the BSF prepupae samples, based on the seasonality of the substrates. In particular, BSF prepupae that had been reared on substrates belonging to the Autumn group are found in correspondence with PC1 positive values, well separated from the others. In this group, the most represented essential amino acids are phenylalanine/tyrosine, valine and leucine.

On the other hand, the All-Year and Summer groups showed a less clear separation between each other and both were found at negative values of PC1. They differed from the Autumn group mainly in their higher amounts of lysine, aspartate and glutamate content. Finally, the control group was found in an intermediate position, closer to the All-Year and Summer groups and with greater differences compared to the Autumn one.

In order to verify to what extent the nutritional properties of BSF prepupae proteins were affected by the composition of the rearing substrates, a one-way ANOVA was carried out on the essential amino acids (EAAs), dividing the samples according to the four groups of substrates (Table 2).

As a general consideration, ANOVA results confirm what is evidenced by PCA; in fact, the Autumn group is the one presenting the larger number of significant differences with respect to the other experimental diets (All-Year and Summer), and also with respect to the control diet. The BSF prepupae of the Autumn group contain the highest amounts of essential amino acids, except for lysine, which was detected in significant lower amounts compared to the BSF reared on the other substrates.

Isoleucine, methionine, cysteine and tryptophan did not differ significantly in any of the four diets.

On the other hand, specific differences in essential amino acid content of prepupae reared on the three diets were observed. Leucine turned out to be the EAA most affected from the diet, highlighting a sharp decline when samples of the All-Year group were considered. A similar trend was also observed for valine and histidine. Phenylalanine and tyrosine were lower in the All-Year and Summer groups when compared with the Autumn group, while threonine in the Summer group was present in a lower amount with respect to the Autumn group. Actually, several peculiar differences were also observed in the single samples (Table S2).

Our results suggested a partial influence of the BSF diet on the total protein production and on their amino acid composition, and similar findings were also obtained by Spranghers et al. [8] and Soetemans et al. [45] on the tenebrionid *Alphitobius diaperinus*. However, it is not yet clear how BSF larvae convert the amino acids from the diet into amino acids useful for their metabolism and how they store and use differently the different essential amino acids. According to Liland et al. [31], BSF larvae were able to produce certain amino acids, such as tyrosine, which were almost absent in the seaweed-containing media.

**Figure 5:**
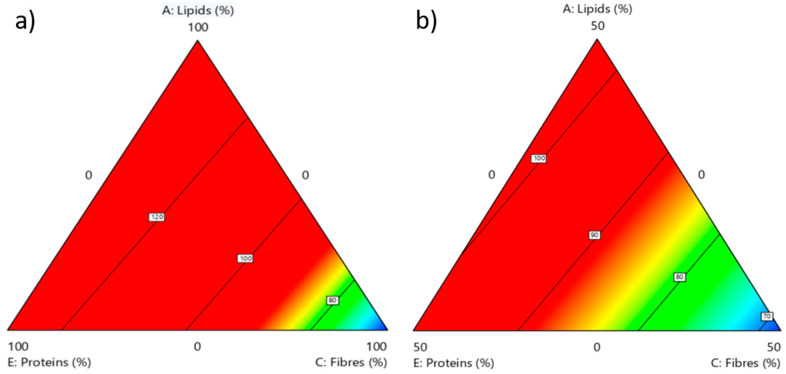
Graphical model variation of the amount of leucine in the proteins of BSF prepupae in relation to the composition of the rearing substrate in terms of lipids, proteins and fibres, considering two scenarios: (**a**) carbohydrates = 0 wt%; (**b**) carbohydrates = 50 wt%. The region representing the highest value of the response is shown in red colour whereas the lowest values are in blue. For each response, the most significant factor has been considered for the graphical model, expecting a higher variation in the response behaviour.

**Figure 6:**
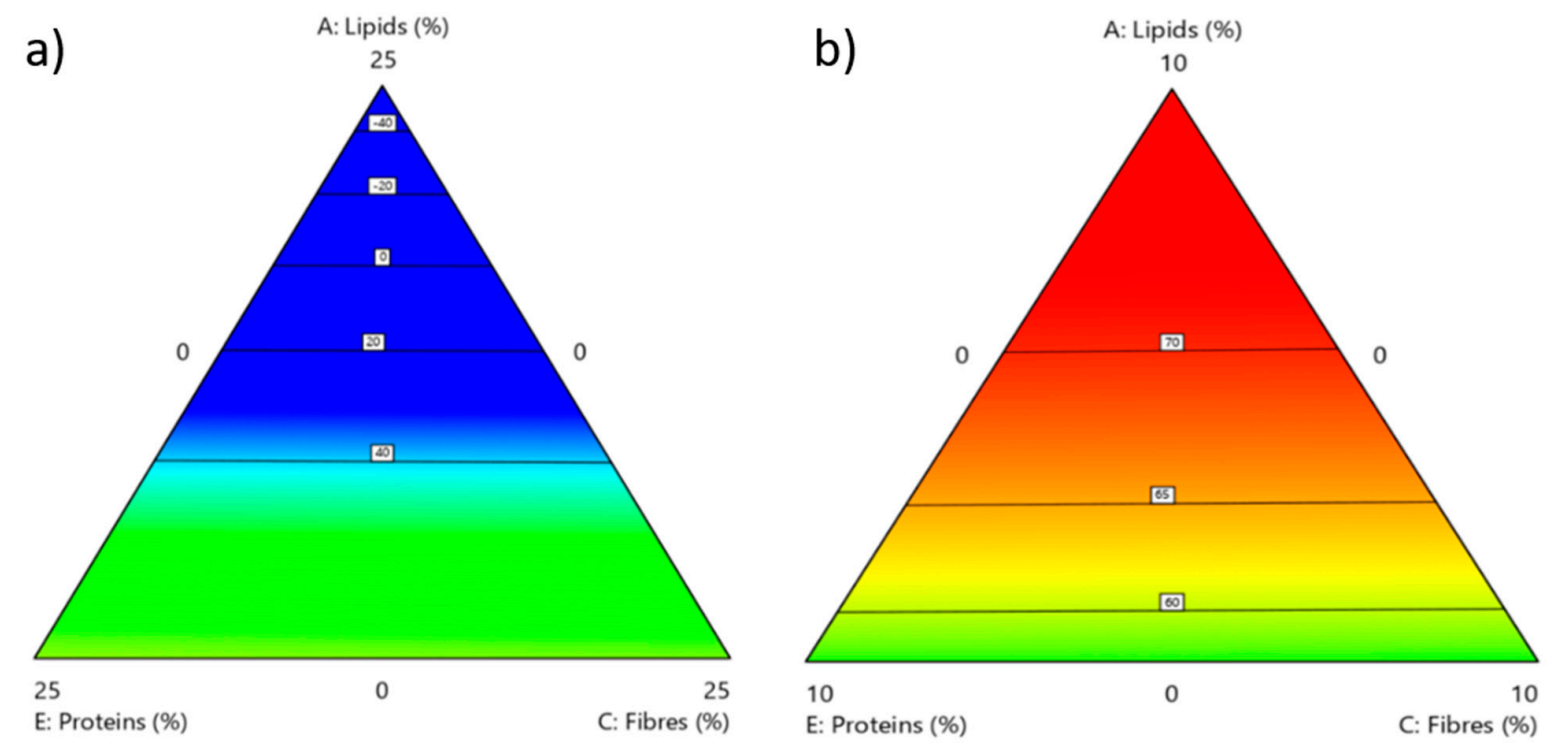
Graphical model variation of the amount of lysine in the proteins of BSF prepupae in relation to the composition of the rearing substrate in terms of lipids, protein and fibre, considering two scenarios: (**a**) carbohydrates = 75 wt%; (**b**) carbohydrates = 90 wt%. The region representing the highest value of the response is shown in red colour, whereas the lowest values are in blue. For each response, the most significant factor has been considered for the graphical model, expecting higher variation in the response behaviour.

Due to the complexity of the system and the possible interaction of diet components on specific essential amino acid content, a multivariate statistical analysis was used as further approach to better understand the influence of the diet composition (Table 1) on the essential amino acid profile. This approach was possible because the experimental diets were formulated according to a Design of Experiments (DoE) [32], allowing us to construct statistically reliable models describing the correlation among food leftovers’ composition and amino acid content, and utilizing ANOVA to verify if the effects of the main factors and their interaction terms are statistically reliable. Results of ANOVA (Table S4) indicate the influence of many significant factors for each response, thus confirming the complex nature of these correlations. These relationships are in general better explained by the interaction between the factors rather than by an independent single factor, confirming the need for the multivariate approach. Furthermore, the fitting parameters show a fairly good fitting (R^2^ > 0.50) only for some responses, in particular for leucine, valine and lysine (Table S4). The graphical representation of the results with acceptable fitting quality is reported in Figure 5 and Figure 6. According to these results, for the leucine content in the BSF prepupae (Figure 5), a strong interaction emerges with the content of lipids and proteins in the rearing substrate. In particular, when the rearing diets were lacking carbohydrates, the highest content of leucine was detected when the content of proteins and lipids in the substrate was equal to or above 50 wt% (Figure 5a). In the presence of a higher content of carbohydrates (50 wt%), the highest content of leucine in BSF prepupae is obtained with slightly lower contents of both proteins and lipids in the rearing diet (Figure 5b). Overall, the increase in carbohydrate content leads to a reduction in the red area, thus indicating that a smaller combination of rearing substrates is suitable for an increase in the leucine content of the BSF prepupae. In conclusion, the highest leucine content in the BSF prepupae can be achieved by increasing the amounts of protein and lipids while reducing the carbohydrates content in the rearing diet. Similarly (data not shown), the valine content in BSF prepupae is enhanced by maximising the content of lipids and proteins and reducing that of carbohydrates in the rearing substrate. The correlation between the content of leucine and valine in the BSF prepupae with the protein content of the rearing substrates could indicate that these amino acids are essential for BSF development.

Finally, the contour plots related to the content of lysine in BSF prepupae (Figure 6) show that carbohydrate variation plays a crucial role in enhancing this amino acid content. In fact, the highest content of lysine in the insects can only be obtained when the rearing substrate has at least 90% DM of carbohydrates (Figure 6b) and a slightly higher amount of lipids compared to protein and fibre. A rearing substrate based on 75% DM of carbohydrates (Figure 6a) will result in prepupae with lower amounts of lysine. In any case, to obtain average values of lysine in the BSF prepupae, in both situations a well-balanced amount of protein and fibre in the rearing substrate is needed.

As a general consideration, it is important to highlight that none of the BSF prepupae reared on experimental diets contained significantly lower amounts of essential amino acids with respect to the control group, except in the above-mentioned case of lysine. Despite the specific differences, the majority of the proteins obtained from the BSF prepupae reared on the experimental substrates contain on average a sufficient quantity of each EAA required for human consumption, as shown in Table 3. Recent studies have shown that BSF prepupae contain optimal amounts of all the essential amino acids (EAA) to satisfy the human adult requirements, as established in the reference of FAO/WHO [7,17,46].

According to these figures, all the analysed BSF prepupae can meet the FAO requirements, and, as in the case of histidine and tryptophan, the EAA content is even double the required amount. Moreover, the lowest values found in the samples turned out to be higher than the recommended amounts, except for lysine, which resulted in being at the lower limit in few cases (two of the BSF samples reared on the Autumn substrates). This finding is particularly relevant, considering that the analysed prepupae samples did not undergo any thermal treatment, which, on the other hand, is very likely to occur when BSF proteins are to be used in food and feed formulations, thus further lowering the lysine amount through the Maillard reaction. Moreover, one factor that could affect the lysine content is the killing method for BSF prepupae [47]. Killing by freezing, which was the method used in this experimental plan, leads to some alteration of the total amino acid fraction, with the notable decrease of lysine and cysteine, likely involved in the process of melanisation, reacting with quinones with their side chain.

## 4. Conclusions

Due to its physiological characteristics and excellent nutritional properties, the BSF is considered one of the most promising candidates in insect farming for feed and food purposes. Aiming at evaluating the effect of the nutrients of the rearing substrate on the protein content and composition of BSF prepupae, we examined 49 different substrates that consisted of variable proportions of different vegetable by-products and were divided into three groups according to their seasonal availability. The results showed that the total protein content of BSF prepupae ranged between 35% and 49% DM, with the highest values in the Autumn group substrates. It has also been observed that a higher protein content in the diet has resulted in a higher prepupae protein content up to a certain value; this vegetable-to-animal protein conversion lowered gradually as dietary proteins were increased, indicating the existence of a minimal critical amount of protein that has to be present in the diet, and that once reached makes any further protein addition to the rearing substrate much less relevant. Dietary fibres also seem to play a positive role in the achievement of BSF prepupae protein biomass. An important outcome of this work focuses on the essential amino acids. Higher amounts of essential amino acids in the BSF prepupae that had been reared with the substrates of the Autumn group were observed. Lysine, leucine and valine were found to be the most correlated with the presence of nutrients of the feeding diet. Leucine and valine were strongly dependent on the content of protein and lipid in the diet, while lysine is correlated to the amount of carbohydrates. Despite these important differences, the essential amino acid composition almost always fully satisfied the FAO nutritional requirements for humans. The only exception was lysine, and only in a very limited number of cases.

In conclusion, this study shows that by providing BSF larvae with substrates based on a very wide range of combinations of vegetable by-products, it is possible to obtain in the BSF prepupae a protein quality very similar to the one obtained with the control diet. However, when the employed leftovers have a very low-quality nutritional content, the development of BSF biomass is less efficient, and as a consequence a lower amount of protein is obtained. Thus, growing on these substrates should be performed by carefully balancing the advantage of re-using leftovers with the disadvantage of having a slightly lower amount of protein. These findings are very important in view of promoting BSF as a flexible tool able to bio-convert a wide range of vegetable by-products, which can vary according to seasonality or areas of production.

## Figures and Tables

**Table 1:** Mean percentage composition of the different diets, expressed as g/100 g dry matter and corresponding amount of total prepupal biomasses obtained (g) starting from 300 Black Soldier Fly (BSF) larvae. Nd = not detected.

	All-Year (n = 21)	Summer (n = 13)	Autumn (n = 15)	CTR
Lipid	0.78 ± 0.35	3.28 ± 2.12	3.18 ± 1.46	3.37 ± 0.1
Ashes	6.66 ± 3.20	4.87 ± 0.71	4.02 ± 1.59	5.9 ± 0.5
Fibres	23.1 ± 17.51	56.86 ± 14.79	56.69 ± 6.53	41 ± 2.5
Polyphenols	0.03 ± 0.02	0.09 ± 0.02	0.04 ± 0.01	Nd
Protein	5.07 ± 1.20	9.97 ± 2.71	16.03 ± 6.57	17.28 ± 1
Available carbohydrates	64.36 ± 20.07	24.93 ± 15.61	17.05 ± 10.72	32.61 ± 1
Total prepupae biomass (g)	21.2 ± 2	39.8 ± 3	57.1 ± 5	52.0 ± 4

**Table 2:** Mean values (expressed as mg/g protein) for essential and semi-essential amino acids of BSF prepupae that had been reared on the different groups of substrates.

	All-Year (n = 21)	Summer (n = 13)	Autumn (n = 15)	CTR (n = 3)
His	33.50 a	37.86 ab	39.00 b	37.00 ab
Ile	42.89 a	43.11 a	44.10 a	45.39 a
Leu	71.24 a	77.66 bc	79.88 c	74.00 ab
Val	60.49 a	60.25 a	66.09 b	62.43 a
Lys	62.22 a	62.93 a	51.73 b	62.44 a
Cys	18.17 a	20.42 a	20.14 a	20.70 a
Met	18.37 a	17.54 a	19.16 a	18.40 a
Phe	41.69 a	40.03 a	46.47 b	42.53 ab
Tyr	61.45 a	63.16 ab	69.67 b	64.13 ab
Thr	39.50 ab	37.36 a	40.58 b	39.30 ab
Trp	14.80 a	15.76 a	15.84 a	17.60 a

Values followed by different letters within one row are significantly different (one-way ANOVA, Tukey post hoc test, *p* < 0.05).

**Table 3:** Highest, lowest and average essential amino acid content of BSF prepupae, compared with the FAO protein reference for human adults (2011).

	Reference Protein FAO 2011 (mg/g Protein)	Average Values Found in BSF Prepupae Protein (mg/g Protein)	Lowest Values Found in BSF Prepupae Protein (mg/g Protein)	Highest Values Found in BSF Prepupae Protein (mg/g Protein)
His	16	36	23	46
Ile	30	44	39	50
Leu	61	76	67	89
Lys	48	58	47	72
Cys + Met	23	38	32	45
Phe + Tyr	41	107	91	127
Thr	25	39	33	44
Trp	6.6	15	7	19
Val	40	62	56	72

## Data Availability

The data presented in this study are available here and in the Supplementary Material.

## Supplementary Materials

The following are available online at https://www.mdpi.com/article/10.3390/foods10081773/s1, Table S1: Formulation and proximate composition of the rearing substrates, and total weight of corresponding prepupae biomass, Table S2: Complete amino acid composition (mg AA/g protein) of BSF prepupae grown on different diets, Table S3: PCA loadings, Table S4: Summary of ANOVA results.
