# Supplementary material for: Effect of the Rearing Substrate on Total Protein and Amino Acid Composition in Black Soldier Fly
Source: Foods. 2021 Jul 30;10(8):1773. doi: 10.3390/foods10081773 (PMC8391998; doi:10.3390/foods10081773)
Supplement: Supplementary file 1 [file foods-10-01773-s001.zip › foods-1244081-supplementary.pdf]

Table S1: Formulation and proximate composition of the rearing substrates, and total weight of corresponding prepupae biomass

	REARING SUBSTRATES FORMULATION											PROXIMATE COMPOSITION OF REARING SUBSTRATES FORMULATED							Total prepupae biomass (g)
	Entomology code	Exotic fruits (%)	Pineapple (%)	Kiwi (%)	Apple (%)	Melon (%)	Peach (%)	Tomato (%)	Legume (%)	Corn (%)	Pomace (%)	Humidity (%)	Lipid (%)	Crude protein (%)	Ash (%)	Polyphenols (%)	Dietary fibre (%)	Non-structural carbohydrate (%)	
All-Year group	A	50	0	0	0	50	/	/	/	/	/	87,51	0,08	0,60	0,60	0,01	3,83	7,72	26,25
	B	10	10	60	10	10	/	/	/	/	/	87,76	0,07	0,77	1,25	0,00	2,43	7,79	19,47
	C	0	100	0	0	0	/	/	/	/	/	86,51	0,21	0,52	0,62	0,00	0,75	11,39	17,15
	D	0	0	0	0	100	/	/	/	/	/	93,78	0,08	0,48	0,71	0,00	5,61	0,00	22,54
	E	0	0	0	100	0	/	/	/	/	/	90,83	0,04	0,35	0,32	0,00	1,54	6,92	20,4
	F	100	0	0	0	0	/	/	/	/	/	81,23	0,08	0,72	0,48	0,01	2,04	15,44	18,31
	G	20	20	20	20	20	/	/	/	/	/	87,98	0,09	0,60	0,77	0,00	2,47	8,22	23,51
	H	50	0	50	0	0	/	/	/	/	/	84,39	0,07	0,83	1,11	0,01	2,22	11,40	20,83
	J	0	50	0	0	50	/	/	/	/	/	90,15	0,15	0,50	0,67	0,00	3,18	5,70	23,44
	L	0	0	50	0	50	/	/	/	/	/	90,66	0,07	0,71	1,22	0,00	4,00	3,68	20,93
	N	0	50	0	50	0	/	/	/	/	/	88,67	0,13	0,44	0,47	0,00	1,15	9,16	22,32
	P	10	10	10	60	10	/	/	/	/	/	89,40	0,07	0,48	0,55	0,00	2,00	7,57	22,77
	Q	0	0	0	50	50	/	/	/	/	/	92,31	0,06	0,42	0,52	0,00	3,58	3,46	24,57
	R	10	10	10	10	60	/	/	/	/	/	90,88	0,09	0,54	0,74	0,00	4,04	4,11	25,34
	S	0	50	50	0	0	/	/	/	/	/	87,03	0,13	0,73	1,18	0,00	1,57	9,38	19,66
	T	50	50	0	0	0	/	/	/	/	/	83,87	0,15	0,62	0,55	0,01	1,40	13,42	20,69
	U	0	0	100	0	0	/	/	/	/	/	87,54	0,05	0,94	1,73	0,00	2,39	7,36	13,06
	V	50	0	0	50	0	/	/	/	/	/	86,03	0,06	0,54	0,40	0,01	1,79	11,18	22,07
	X	60	10	10	10	10	/	/	/	/	/	84,60	0,09	0,66	0,63	0,01	2,25	11,83	22,59
	Y	10	60	10	10	10	/	/	/	/	/	87,24	0,15	0,56	0,70	0,00	1,61	9,81	20,6
	Z	0	0	50	50	0	/	/	/	/	/	89,19	0,05	0,65	1,03	0,00	1,97	7,14	19,59
	Entomology code	Exotic fruits (%)	Pineapple (%)	Kiwi (%)	Apple (%)	Melon (%)	Peach (%)	Tomato (%)	Legume (%)	Corn (%)	Pomace (%)	Humidity (%)	Lipid (%)	Crude protein (%)	Ash (%)	Polyphenols (%)	Dietary fibre (%)	Non-structural carbohydrate (%)	
Summer group	A	0	/	/	/	0	67	33	/	/	/	86,48	0,67	1,59	0,62	0,02	8,13	2,87	39,54
	B	0	/	/	/	0	100	0	/	/	/	89,89	0,82	0,87	0,52	0,01	3,58	4,30	36,69
	C	20	/	/	/	47	16	17	/	/	/	88,27	0,25	1,01	0,65	0,01	6,49	3,81	36,9
	D	10	/	/	/	24	33	33	/	/	/	86,53	0,43	1,48	0,66	0,01	8,45	2,98	39,45
	E	10	/	/	/	23	0	67	/	/	/	83,12	0,28	2,20	0,76	0,02	13,00	1,54	42,42
	F	20	/	/	/	47	33	0	/	/	/	89,97	0,33	0,66	0,60	0,01	4,22	4,52	36,33

	G	5	/	/	/	12	67	16	/	/	/	88,21	0,62	1,18	0,59	0,01	6,01	3,64	37,21
	H	20	/	/	/	47	0	33	/	/	/	86,57	0,18	1,37	0,70	0,01	8,77	3,09	36,37
	I	30	/	/	/	70	0	0	/	/	/	90,02	0,08	0,55	0,64	0,00	4,54	4,63	35,01
	J	0	/	/	/	0	33	67	/	/	/	83,08	0,52	2,31	0,72	0,02	12,68	1,43	41,89
	K	5	/	/	/	11	17	67	/	/	/	83,10	0,40	2,25	0,74	0,02	12,84	1,49	41,01
	L	0	/	/	/	0	0	100	/	/	/	79,68	0,37	3,02	0,82	0,02	17,23	0,00	50,46
	M	10	/	/	/	23	67	0	/	/	/	89,93	0,58	0,76	0,56	0,01	3,90	4,41	39,52
	Entomology code	Exotic fruits (%)	Pineapple (%)	Kiwi (%)	Apple (%)	Melon (%)	Peach (%)	Tomato (%)	Legume (%)	Corn (%)	Pomace (%)	Humidity (%)	Lipid (%)	Crude protein (%)	Ash (%)	Polyphenols (%)	Dietary fibre (%)	Non-structural carbohydrate (%)	
Autumn group	A	4	/	/	/	8,5	/	/	12,5	62,5	12,5	60,37	1,23	8,51	1,06	0,01	26,47	2,40	62,88
	B	15	/	/	/	35	/	/	0	50	0	71,11	0,76	6,02	0,80	0,01	19,06	2,47	67,4
	C	0	/	/	/	0	/	/	0	100	0	52,20	1,44	11,49	0,97	0,01	33,59	0,30	51,47
	D	4	/	/	/	8,5	/	/	12,5	12,5	62,5	69,94	1,43	3,49	1,57	0,01	18,01	5,60	52,14
	E	4	/	/	/	8,5	/	/	62,5	12,5	12,5	64,59	0,86	7,09	1,11	0,01	20,99	5,41	60,95
	F	0	/	/	/	0	/	/	100	0	0	60,63	0,70	8,65	1,05	0,01	22,63	6,33	48,08
	G	0	/	/	/	0	/	/	50	0	50	65,98	1,28	5,05	1,52	0,01	19,64	6,52	55,75
	H	30	/	/	/	70	/	/	0	0	0	90,02	0,08	0,55	0,64	0,00	4,54	4,63	40,60
	I	0	/	/	/	0	/	/	50	50	0	56,42	1,07	10,07	1,01	0,01	28,11	3,32	54,33
	J	15	/	/	/	35	/	/	0	0	50	80,67	0,96	1,00	1,31	0,01	10,60	5,67	40,64
	K	19	/	/	/	43,5	/	/	12,5	12,5	12,5	79,28	0,55	3,04	0,90	0,01	11,95	4,56	55,55
	L	0	/	/	/	0	/	/	0	0	100	71,33	1,85	1,45	1,98	0,02	16,66	6,71	18,46
	M	0	/	/	/	0	/	/	0	50	50	61,76	1,64	6,47	1,47	0,01	25,13	3,51	58,68
	N	15	/	/	/	35	/	/	50	0	0	75,32	0,39	4,60	0,85	0,01	13,58	5,48	56,91
	O	7,5	/	/	/	17,5	/	/	25	25	25	68,54	1,02	5,53	1,16	0,01	19,35	4,49	65,64

Table S2: Complete amino acid composition (mg AA/g protein) of BSF prepupae grown on different diets

		mg AA/g protein																	
		ala	asp + asn	arg	gly	his	ile	leu	met	phe	pro	ser	thr	val	lys	tyr	glu + gln	trp	cys
	Code																		
ALL-YEAR GROUP	A	65 ± 6	113 ± 14	69 ± 8	60 ± 7	23 ± 16	42 ± 2	67 ± 2	19 ± 1	42 ± 7	72 ± 8	53 ± 2	41 ± 0	60 ± 2	60 ± 13	61 ± 14	124 ± 1	14 ± 2	14 ± 0
	B	62 ± 7	114 ± 16	71 ± 18	50 ± 0	33 ± 5	42 ± 1	70 ± 0	19 ± 0	37 ± 5	73 ± 5	48 ± 1	40 ± 2	60 ± 1	59 ± 8	56 ± 7	131 ± 18	18 ± 1	18 ± 0
	C	62 ± 2	92 ± 19	80 ± 7	57 ± 7	37 ± 13	43 ± 2	69 ± 4	19 ± 0	48 ± 12	69 ± 2	50 ± 3	41 ± 3	60 ± 1	59 ± 15	73 ± 16	108 ± 19	16 ± 3	17 ± 2
	D	63 ± 2	106 ± 2	72 ± 10	56 ± 0	30 ± 1	42 ± 1	70 ± 0	22 ± 2	49 ± 0	68 ± 4	49 ± 0	41 ± 1	61 ± 1	57 ± 8	69 ± 11	109 ± 8	17 ± 0	17 ± 1
	E	65 ± 3	118 ± 5	71 ± 12	51 ± 3	36 ± 5	41 ± 2	69 ± 1	17 ± 1	38 ± 1	70 ± 4	47 ± 2	39 ± 4	59 ± 3	61 ± 2	59 ± 3	126 ± 2	15 ± 1	19 ± 2
	F	65 ± 2	111 ± 9	70 ± 5	51 ± 1	35 ± 5	45 ± 1	72 ± 4	18 ± 1	39 ± 2	72 ± 1	49 ± 3	38 ± 0	60 ± 3	64 ± 8	58 ± 0	120 ± 0	14 ± 0	17 ± 2
	G	72 ± 12	107 ± 18	63 ± 2	54 ± 7	27 ± 4	46 ± 5	75 ± 9	17 ± 0	40 ± 1	71 ± 3	46 ± 6	36 ± 8	66 ± 4	70 ± 8	51 ± 10	128 ± 7	14 ± 1	16 ± 0
	H	61 ± 4	105 ± 6	78 ± 1	53 ± 2	33 ± 0	43 ± 2	71 ± 2	18 ± 1	43 ± 1	69 ± 3	50 ± 2	38 ± 1	60 ± 1	63 ± 1	61 ± 4	121 ± 4	15 ± 1	19 ± 3
	J	64 ± 3	103 ± 8	75 ± 3	54 ± 10	37 ± 2	43 ± 2	72 ± 1	18 ± 5	42 ± 6	72 ± 2	56 ± 7	38 ± 0	60 ± 3	59 ± 15	66 ± 13	117 ± 16	7 ± 1	16 ± 2
	L	66 ± 3	115 ± 2	74 ± 4	54 ± 9	34 ± 5	39 ± 3	71 ± 3	16 ± 1	35 ± 3	72 ± 3	47 ± 2	35 ± 2	59 ± 5	64 ± 17	56 ± 9	128 ± 8	15 ± 1	20 ± 1
	N	67 ± 3	110 ± 5	73 ± 2	47 ± 1	31 ± 3	45 ± 4	73 ± 6	16 ± 0	39 ± 5	71 ± 1	47 ± 1	38 ± 1	63 ± 4	72 ± 3	53 ± 1	129 ± 14	12 ± 2	16 ± 4
	P	66 ± 2	109 ± 7	71 ± 7	50 ± 2	33 ± 3	44 ± 1	71 ± 0	20 ± 1	42 ± 3	68 ± 3	50 ± 0	42 ± 2	63 ± 1	60 ± 6	62 ± 1	116 ± 5	17 ± 2	15 ± 3
	Q	63 ± 3	106 ± 3	78 ± 6	50 ± 3	37 ± 5	43 ± 1	73 ± 3	21 ± 1	41 ± 1	64 ± 0	50 ± 2	40 ± 0	60 ± 0	60 ± 4	59 ± 3	118 ± 4	19 ± 1	19 ± 2
	R	64 ± 4	106 ± 3	77 ± 2	50 ± 3	33 ± 5	41 ± 2	72 ± 2	18 ± 0	42 ± 2	68 ± 4	49 ± 3	40 ± 0	58 ± 0	65 ± 2	63 ± 5	119 ± 1	15 ± 1	21 ± 3
	S	64 ± 4	108 ± 1	74 ± 5	51 ± 3	33 ± 0	42 ± 1	71 ± 3	17 ± 2	42 ± 1	68 ± 4	49 ± 3	40 ± 1	59 ± 2	61 ± 10	63 ± 1	119 ± 1	16 ± 0	22 ± 0
	T	65 ± 1	111 ± 6	73 ± 0	51 ± 2	35 ± 1	43 ± 1	71 ± 0	18 ± 1	41 ± 2	64 ± 1	48 ± 2	41 ± 1	62 ± 1	64 ± 8	63 ± 3	116 ± 7	14 ± 0	20 ± 3
	U	62 ± 2	100 ± 5	68 ± 2	50 ± 5	31 ± 3	44 ± 0	73 ± 2	19 ± 2	45 ± 11	72 ± 2	53 ± 3	42 ± 1	61 ± 1	61 ± 17	63 ± 15	125 ± 12	12 ± 0	20 ± 1
	V	61 ± 1	105 ± 9	79 ± 3	48 ± 1	38 ± 1	42 ± 1	71 ± 0	19 ± 0	42 ± 1	67 ± 4	51 ± 4	40 ± 0	57 ± 0	62 ± 3	60 ± 2	122 ± 7	16 ± 2	19 ± 1
	X	60 ± 2	101 ± 15	82 ± 6	51 ± 6	36 ± 2	44 ± 3	72 ± 1	19 ± 1	47 ± 5	67 ± 3	47 ± 2	39 ± 0	61 ± 4	55 ± 8	70 ± 11	110 ± 0	18 ± 4	20 ± 2
	Y	61 ± 6	105 ± 12	80 ± 1	49 ± 2	38 ± 7	44 ± 1	72 ± 1	18 ± 2	42 ± 7	68 ± 4	49 ± 2	40 ± 2	61 ± 1	62 ± 12	66 ± 9	114 ± 9	12 ± 1	17 ± 2
	Z	63 ± 4	104 ± 8	77 ± 6	48 ± 3	33 ± 5	43 ± 3	72 ± 0	18 ± 0	38 ± 5	71 ± 0	50 ± 4	39 ± 5	60 ± 4	69 ± 3	55 ± 7	125 ± 5	14 ± 1	21 ± 2

		ala	asp + asn	arg	gly	his	ile	leu	met	phe	pro	ser	thr	val	lys	tyr	glu + gln	trp	cys
	Entomology code																		
SUM	A	64 ± 3	96 ± 1	76 ± 6	48 ± 3	41 ± 2	44 ± 1	75 ± 1	18 ± 1	42 ± 5	70 ± 0	46 ± 2	36 ± 2	59 ± 2	66 ± 11	62 ± 4	118 ± 3	19 ± 1	20 ± 2

	B	67 ± 3	91 ± 3	77 ± 2	47 ± 1	41 ± 2	46 ± 0	84 ± 8	15 ± 2	38 ± 3	71 ± 4	44 ± 0	33 ± 1	60 ± 2	63 ± 4	61 ± 0	122 ± 1	17 ± 3	24 ± 5
	C	65 ± 2	97 ± 1	84 ± 2	47 ± 2	38 ± 0	44 ± 4	80 ± 1	17 ± 1	40 ± 3	68 ± 1	45 ± 0	35 ± 0	59 ± 1	66 ± 5	61 ± 5	115 ± 1	19 ± 5	22 ± 0
	D	62 ± 3	101 ± 5	73 ± 8	50 ± 3	41 ± 2	44 ± 2	79 ± 4	19 ± 1	40 ± 0	66 ± 2	45 ± 2	38 ± 5	60 ± 2	67 ± 9	61 ± 1	120 ± 2	15 ± 3	19 ± 3
	E	64 ± 2	103 ± 1	73 ± 1	47 ± 3	38 ± 0	43 ± 3	77 ± 2	18 ± 1	41 ± 0	70 ± 6	45 ± 3	38 ± 2	62 ± 2	64 ± 7	61 ± 2	122 ± 4	14 ± 0	19 ± 1
	F	66 ± 2	94 ± 1	73 ± 7	51 ± 2	46 ± 0	41 ± 1	75 ± 1	18 ± 0	42 ± 1	73 ± 2	47 ± 1	39 ± 1	57 ± 0	57 ± 2	64 ± 4	117 ± 6	16 ± 1	23 ± 2
	G	65 ± 3	94 ± 8	77 ± 5	52 ± 3	39 ± 3	41 ± 0	76 ± 1	17 ± 3	45 ± 7	71 ± 2	50 ± 2	40 ± 1	56 ± 2	54 ± 4	75 ± 12	114 ± 9	13 ± 1	22 ± 3
	H	66 ± 1	107 ± 4	75 ± 5	46 ± 3	31 ± 6	43 ± 1	76 ± 1	18 ± 1	39 ± 3	70 ± 2	46 ± 2	37 ± 0	58 ± 0	61 ± 12	62 ± 4	124 ± 2	17 ± 3	23 ± 2
	I	62 ± 2	100 ± 2	71 ± 8	45 ± 2	38 ± 8	41 ± 3	76 ± 3	18 ± 2	41 ± 3	69 ± 1	46 ± 4	40 ± 4	62 ± 2	67 ± 3	63 ± 0	127 ± 6	15 ± 3	19 ± 1
	J	57 ± 4	99 ± 6	75 ± 1	50 ± 0	32 ± 7	40 ± 3	81 ± 7	16 ± 1	36 ± 5	77 ± 8	47 ± 0	37 ± 4	64 ± 1	59 ± 5	62 ± 1	134 ± 4	16 ± 1	17 ± 2
	K	65 ± 0	99 ± 6	79 ± 10	49 ± 1	34 ± 3	44 ± 4	78 ± 0	19 ± 3	41 ± 2	68 ± 1	44 ± 1	36 ± 3	61 ± 3	61 ± 12	66 ± 3	122 ± 2	14 ± 2	21 ± 7
	L	65 ± 1	104 ± 4	66 ± 4	53 ± 2	36 ± 3	46 ± 2	76 ± 1	19 ± 3	37 ± 2	67 ± 2	41 ± 2	38 ± 0	66 ± 2	70 ± 1	64 ± 3	120 ± 0	14 ± 0	17 ± 0
	M	68 ± 3	104 ± 4	72 ± 3	47 ± 3	36 ± 3	43 ± 1	77 ± 4	17 ± 1	39 ± 4	71 ± 0	46 ± 4	37 ± 1	61 ± 1	64 ± 11	58 ± 11	124 ± 9	16 ± 2	20 ± 0

		**ala**	**asp + asn**	**arg**	**gly**	**his**	**ile**	**leu**	**me t**	**phe**	**pro**	**ser**	**thr**	**val**	**lys**	**tyr**	**glu + gln**	**trp**	**cys**
	Entomo logy code																		
**AUTUMN GROUP**	A	64 ± 2	83 ± 1	82 ± 12	59 ± 4	41 ± 2	44 ± 2	83 ± 3	21 ± 2	51 ± 3	73 ± 2	50 ± 9	40 ± 8	66 ± 3	55 ± 7	76 ± 0	87 ± 2	19 ± 0	23 ± 1
	B	68 ± 3	91 ± 8	77 ± 3	61 ± 1	42 ± 5	44 ± 1	77 ± 3	20 ± 0	50 ± 1	74 ± 3	56 ± 2	42 ± 4	66 ± 2	47 ± 1	73 ± 2	90 ± 4	16 ± 2	21 ± 3
	C	64 ± 1	94 ± 3	77 ± 6	57 ± 2	38 ± 3	45 ± 0	81 ± 1	20 ± 2	48 ± 6	69 ± 5	58 ± 4	42 ± 5	68 ± 1	66 ± 8	68 ± 8	86 ± 1	14 ± 2	17 ± 1
	D	68 ± 2	79 ± 11	86 ± 10	61 ± 7	37 ± 1	44 ± 2	78 ± 5	20 ± 1	49 ± 5	81 ± 6	48 ± 8	37 ± 2	64 ± 3	47 ± 6	78 ± 5	97 ± 14	17 ± 0	24 ± 2
	E	66 ± 2	89 ± 4	78 ± 5	58 ± 6	39 ± 4	48 ± 2	81 ± 3	19 ± 1	48 ± 4	76 ± 3	55 ± 2	40 ± 2	67 ± 2	50 ± 11	71 ± 7	90 ± 1	13 ± 5	21 ± 0
	F	69 ± 3	95 ± 9	68 ± 6	55 ± 2	35 ± 2	48 ± 1	89 ± 3	19 ± 1	42 ± 1	69 ± 1	56 ± 5	43 ± 4	69 ± 2	63 ± 8	62 ± 4	96 ± 6	13 ± 1	15 ± 0
	G	62 ± 5	89 ± 5	75 ± 8	57 ± 4	38 ± 4	50 ± 1	79 ± 1	18 ± 2	44 ± 2	77 ± 4	51 ± 4	40 ± 2	67 ± 4	51 ± 4	69 ± 6	101 ± 10	15 ± 1	19 ± 2
	H	63 ± 2	95 ± 26	83 ± 13	55 ± 2	42 ± 2	42 ± 2	73 ± 1	20 ± 0	46 ± 1	72 ± 2	56 ± 11	37 ± 2	61 ± 1	54 ± 5	70 ± 3	102 ± 2	16 ± 1	21 ± 3
	I	68 ± 2	93 ± 2	73 ± 6	56 ± 1	40 ± 2	47 ± 1	84 ± 1	19 ± 1	46 ± 3	70 ± 0	55 ± 6	43 ± 1	67 ± 3	55 ± 6	67 ± 1	88 ± 2	15 ± 1	18 ± 0
	J	66 ± 4	85 ± 4	80 ± 3	55 ± 2	35 ± 1	43 ± 3	74 ± 1	18 ± 1	43 ± 2	79 ± 1	49 ± 3	35 ± 1	59 ± 2	60 ± 10	72 ± 1	113 ± 0	18 ± 1	23 ± 1
	K	76 ± 7	99 ± 2	70 ± 6	53 ± 1	37 ± 2	45 ± 2	79 ± 1	18 ± 0	43 ± 2	68 ± 1	53 ± 0	44 ± 8	66 ± 0	59 ± 7	60 ± 1	102 ± 6	15 ± 1	19 ± 1
	L	64 ± 5	76 ± 8	88 ± 7	55 ± 2	39 ± 2	42 ± 1	73 ± 1	19 ± 2	48 ± 5	81 ± 3	46 ± 1	38 ± 2	64 ± 2	51 ± 9	70 ± 6	107 ± 1	18 ± 0	26 ± 2
	M	65 ± 1	83 ± 16	73 ± 6	62 ± 6	40 ± 2	49 ± 4	86 ± 2	18 ± 2	46 ± 4	76 ± 4	55 ± 0	44 ± 3	72 ± 0	49 ± 9	71 ± 6	87 ± 11	14 ± 1	17 ± 0
	N	65 ± 3	85 ± 13	77 ± 11	57 ± 3	43 ± 7	43 ± 1	79 ± 5	19 ± 0	47 ± 3	73 ± 1	55 ± 5	42 ± 6	67 ± 1	56 ± 5	69 ± 1	92 ± 3	16 ± 2	20 ± 1
	O	68 ± 2	89 ± 11	73 ± 5	58 ± 4	40 ± 3	46 ± 1	80 ± 2	18 ± 1	47 ± 1	74 ± 2	54 ± 4	42 ± 1	69 ± 0	51 ± 1	68 ± 2	88 ± 1	17 ± 2	18 ± 1

		**ala**	**asp + asn**	**arg**	**gly**	**his**	**ile**	**leu**	**me t**	**phe**	**pro**	**ser**	**thr**	**val**	**lys**	**tyr**	**glu + gln**	**trp**	**cys**
**CONTROL**	Entomology code																		
	CTR	66 ± 2	99 ± 9	77 ± 9	51 ± 1	37 ± 1	44 ± 2	74 ± 4	18 ± 0	43 ± 3	69 ± 0	47 ± 2	39 ± 0	62 ± 3	62 ± 6	64 ± 3	109 ± 5	18 ± 2	21 ± 3

Table S3: PCA loadings.

	PC1	PC2	PC3	PC4	PC5
glu	-0,94	-0,14	-0,01	-0,20	0,04
lys	-0,90	0,16	0,08	0,19	-0,13
phe	0,86	-0,08	-0,34	0,08	0,03
asp	-0,84	0,33	-0,30	0,05	0,09
tyr	0,82	-0,31	-0,23	-0,07	-0,11
gly	0,80	0,23	-0,18	-0,30	0,26
val	0,67	0,53	0,30	0,02	0,07
ser	0,62	0,46	-0,30	-0,17	-0,19
his	0,52	-0,36	0,23	0,31	-0,48
cys	0,22	-0,82	0,22	0,08	0,06
arg	0,38	-0,76	-0,16	-0,01	-0,13
thr	0,43	0,62	-0,39	0,22	-0,14
trp	0,14	-0,53	0,00	0,47	0,50
leu	0,54	0,15	0,67	0,15	-0,22
met	0,54	0,05	-0,60	0,33	0,23
ile	0,47	0,45	0,50	0,15	0,03
pro	0,50	-0,24	0,25	-0,70	0,20
ala	0,17	0,30	0,48	0,18	0,50

Table S4. Summary of ANOVA results.

Response	Transformation	Significant factors other than linear factors	R^2	Pred R^2
his	None	Ashes*Fibres*Carbohydrates<br>Ashes*Fibres<br>Ashes*Carbohydrates<br>Fibres*Carbohydrates	0.39	0.25
	= 115,1251 * Lipids + 142,8697 * Ashes + 38,8390 * Fibres - 1685,9516 * Polyphenols + 43,4625 * Proteins + 39,3171 * Carbohydrates -250,6740 * Ashes * Fibres -272,3182 * Ashes * Carbohydrates - 9,7396 * Fibres * Carbohydrates + 506,4663 * Ashes * Fibres * Carbohydrates			
ile	None	Ashes* Proteins	0.42	0.34
	= 74,8870 * Lipids - 40,7818 *Ashes + 43,3120 * Fibres -1796,7832 * Polyphenols+ 37,9499 * Proteins + 46,5295 * Carbohydrates + 972,5378 *Ashes * Proteins			
leu	Power (k=2.5)	None	0.76	0.73
	= 138,8949 * Lipids + 56,4144 * Ashes + 67,6174 * Fibres + 1404,0497 * Polyphenols + 128,6415 * Proteins + 68,7274 * Carbohydrates			
met	None	None	0.38	0.30
	= -0,1543 * Lipids + 8,1371 * Ashes + 22,8876 * Fibres -1975,3666 * Polyphenols + 15,3176 * Proteins + 19,1364 * Carbohydrates			
phe	None	Lipids*Fibres	0.51	0.44
	= -40,2706 * Lipids + 20,9231 * Ashes + 44,2497 * Fibres - 8333,6909 * Polyphenols + 51,8682 * Proteins + 46,1981 * Carbohydrates + 371,7611 * Lipids * Fibres			
thr	None	None	0.47	0.41
	= 20,2967 * Lipids + 29,0736 * Ashes + 39,5218 * Fibres -3503,6628 * Polyphenols + 59,5214 * Proteins + 40,8414 * Carbohydrates			
val	None	Lipids * Carbohydrates<br>Polyphenols * Proteins	0.68	0.62
	= 207,4451 * Lipids + 38,1122 * Ashes + 60,6184 * Fibres -11825,8780 * Polyphenols + 74,0273 * Proteins + 66,4548 * Carbohydrates -304,7381 * Lipids * Carbohydrates + 75299,2771 *Polyphenols * Proteins			
lys	Power (k=2.5)	Lipids * Ashes * Polyphenols<br>Lipids * Ashes<br>Lipids * Polyphenols<br>Lipids * Carbohydrates<br>Ashes * Polyphenols	0.63	0.52

	-789,5534 * Lipids + 67,3562 * Ashes + 63,5633 * Fibres + 5323,1377 * Polyphenols + 65,4436 * Proteins + 56,3629 * Carbohydrates + 3172,7599 *Lipids * Ashes + 244403,0963 * Lipids * Polyphenols - 455,9764 *Lipids * Carbohydrates + 18499,4861 * Ashes * Polyphenols + 4911650,4178 * Lipids * Ashes * Polyphenols			
trp	None	Polyphenols * Proteins	0.19	0.08
	= 21,8816 * Lipids + 11,1148 * Ashes + 16,4850 * Fibres + 7629,3982 * Polyphenols + 23,4770 * Proteins + 11,7696 * Carbohydrates -71645,1372 * Polyphenols * Proteins			
